# Exploiting locational and topological overlap model to identify modules in protein interaction networks

**DOI:** 10.1186/s12859-019-2598-7

**Published:** 2019-01-14

**Authors:** Lixin Cheng, Pengfei Liu, Dong Wang, Kwong-Sak Leung

**Affiliations:** 1 Department of Computer Science and Engineering, The Chinese University of Hong Kong, Shatin, Hong Kong; 2 Institute of translation medicine, Shenzhen Second People’s Hospital, First Affiliated Hospital of Shenzhen University, Shenzhen, China; 3 Department of Bioinformatics, School of Basic Medical Sciences, Southern Medical University, Guangzhou, China

**Keywords:** Protein interaction network, Network clustering, Subcellular localization, Topological overlap, Functional module

## Abstract

**Background:**

Clustering molecular network is a typical method in system biology, which is effective in predicting protein complexes or functional modules. However, few studies have realized that biological molecules are spatial-temporally regulated to form a dynamic cellular network and only a subset of interactions take place at the same location in cells.

**Results:**

In this study, considering the subcellular localization of proteins, we first construct a co-localization human protein interaction network (PIN) and systematically investigate the relationship between subcellular localization and biological functions. After that, we propose a Locational and Topological Overlap Model (LTOM) to preprocess the co-localization PIN to identify functional modules. LTOM requires the topological overlaps, the common partners shared by two proteins, to be annotated in the same localization as the two proteins. We observed the model has better correspondence with the reference protein complexes and shows more relevance to cancers based on both human and yeast datasets and two clustering algorithms, ClusterONE and MCL.

**Conclusion:**

Taking into consideration of protein localization and topological overlap can improve the performance of module detection from protein interaction networks.

**Electronic supplementary material:**

The online version of this article (10.1186/s12859-019-2598-7) contains supplementary material, which is available to authorized users.

## Background

Biological networks have received much attention over the last two decades because they systematically model the complex interactions occurring among different components in cells [1–6]. Protein Interaction Network (PIN) is the most common biological networks where the cellular components are proteins [1, 6]. Specifically, the nodes correspond to proteins and the edges correspond to interactions between proteins. Interacting protein pairs often participate in the same biological processes or associate with specific molecular functions. In system biology, clustering PIN is a typical and effective operation to predict protein complexes or functional modules, where a module is a cluster of densely connected proteins in a PIN. The detection of modules using biological networks can help in understanding the mechanisms regulating cell life and predicting the biological functions of the uncharacterized proteins [7–11].

This type of problems can be computationally addressed using clustering techniques and quite a number of approaches are available [7–9]. However, practically all the existing clustering models emerge from analysis at the global cellular level, leading to challenges when considering the context of subcellular localization, as the protein interactions take place in the same locations of cells [5, 6]. For example, eukaryotic cells are organized into a number of compartments that are specialized for various biological functions. *Park* et al. indicated that erroneous localization of proteins is able to result in cell abnormality and human disease [12]. Furthermore, proteins change their localizations frequently and it is an efficient regulation mode in cells. A good example is the pivotal cancer gene, p53, which mainly functions as a transcriptional factor when localized in the nucleus, while upon stimulation it activates several of cellular programs including autophagy, a cellular process of self-eating [13, 14]. In contrast, when translocated from nucleus to cytoplasm, p53 amazingly acts as a master repressor of autophagy [15, 16]. These biological facts are hard to be reflected based on the analysis of the global cellular network, but rather through exploring the co-localization protein interaction networks.

While the protein pairs are generally regarded as independent in a PIN, the topological overlap not only considers the direct interaction between proteins but also considers their indirect connections with all the other proteins in the network [17, 18]. Specifically, a high topological overlap between a pair of proteins refers to they share a large fraction of partners in the network. It has been well studied that two substrates with a high topological overlap tend to be functionally similar [19, 20]. However, in the co-localization protein interaction network, the overlapping partner of the interacting proteins may not have the same localization as them. For instance, a common partner may share a location (such as nucleus) with one protein while belonging to another location (such as membrane) of the other one. Hence, the overlapping partners of two interacting proteins should be annotated in locations the same as the two interacting proteins.

In this study, we first constructed a co-localization protein interaction network (CLPIN) and demonstrated that proteins in the CLPIN are engaged in interactions with high experimental confidence. Then, we applied the proposed Locational and Topological Overlap Model (LTOM) and its old version to impute the missing interactions of CLPIN, producing two new networks, Locational and Topological Overlap PIN (LTOPIN) and Topological Overlap PIN (TOPIN), respectively. Our results show that the LTOM inferred network outperforms the counterparts in module identification based on the human and yeast datasets and two clustering methods, ClusterONE and MCL. Finally, the biological functions of the identified modules were further investigated by associating with human cancer genes.

## Materials and methods

### Subcellular localization information

The information of protein localization in cells was obtained from the Universal Protein Resource (UniProt) [21, 22]. It contains 15,950 proteins and 20,565 interaction relationships in 12 subcellular locations, i.e., extracellular, plasma membrane, cytoplasm, mitochondria, Golgi apparatus, endoplasmic reticulum, endosome, peroxisome, lysosome, vacuole, vesicles, and nucleus. The same as Veres et al., we merged mitochondria, Golgi apparatus, endoplasmic reticulum, endosome, peroxisome, lysosome, vacuole and vesicles into a major location “secretary-pathway” [23]. As a result, proteins were efficiently annotated to six major subcellular localizations, i.e., nucleus, cytoplasm, membrane, extracellular, mitochondria, and secretary-pathway.

### Protein interaction networks

Both human and yeast protein-protein interaction (PPI) datasets were studied in this study. The non-redundant relationship of human PPIs was collected from the Human Protein Reference Database (HPRD, Release 9) [24] and the Biological General Repository for Interaction Datasets (BioGRID, 3.4.150) [25], respectively. HPRD is a well-accepted resource of curated proteomic information including only experimental verified interactions. BioGRID is an interaction repository with data compiled through comprehensive curation efforts. As described in Yong et al. [26], the yeast PPIs were obtained by incorporating three databases, BioGRID, IntAct, and MINT. Hereafter, global protein interaction network (GPIN) was used to represent the original PPI networks without filtering interactions.

**Fig. 1:**
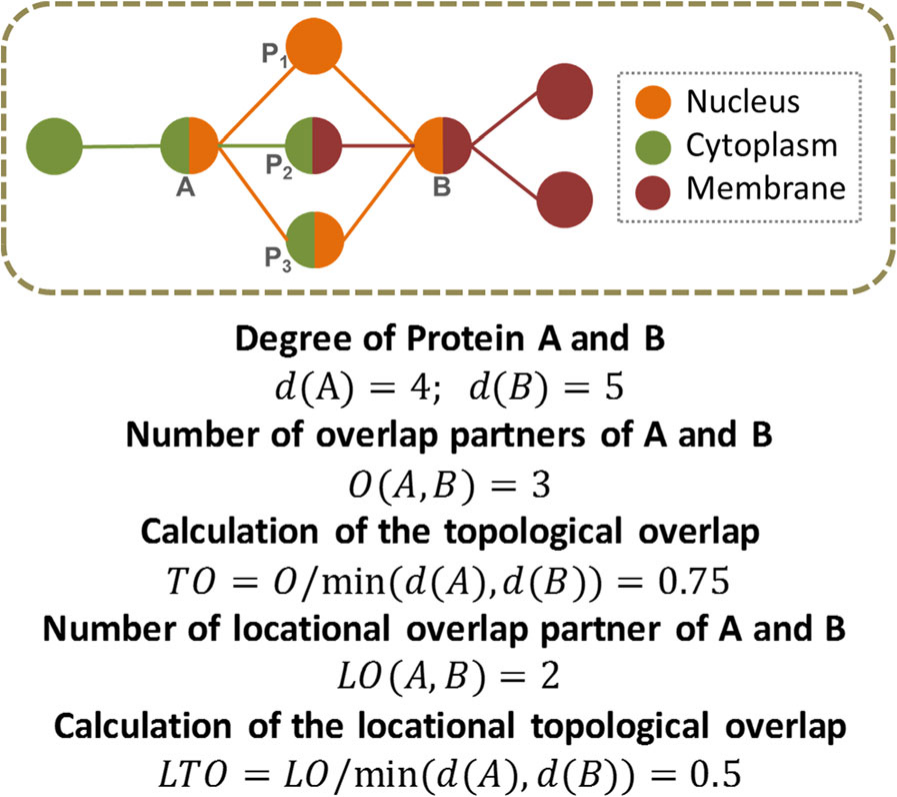
The flowchart of module identification. For a given protein interaction network, GPIN, it is first filtered to CLPIN by the context of cell localization, and then it is imputed to form LTOPIN using both the locational and topological information. After that, ClusterONE is used to identify modules. Finally, the modules are evaluated using three functional categories, biological process, molecular function and cancer gene set. LTOM, Locational and Topological Overlap Model; *Sn*, Sensitivity; *PPV*, Positive Predictive Value; *Acc*, Accuracy

Then, we obtained the co-localization protein interaction networks (CLPIN) by integrating each GPIN with the subcellular localization information. The interacting protein pairs in CLPIN were required to share at least one location and the interactions do not meet the requirement were filtered out. After that, Topological Overlap PIN (TOPIN) and Locational and Topological Overlap PIN (LTOPIN) were constructed based on CLPIN, but only potential interactions were added in and no extra external proteins were recruited, so they contain the same number of proteins as CLPIN. Please see more details in the following sections and Fig. 1.

### The topological overlap matrix model (TOM)

In this part, we introduce the model of Topological Overlap Matrix (TOM) based on the work of Yip et al. [8]. The idea behind TOM is that given the direct interaction information between proteins, we can predict the indirect interaction between proteins by counting the shared intermedia between them. Suppose *G* is the one-step adjacency matrix, and its element *a*_*ij*_ = 1 when there is an interaction between protein *i* and protein *j*, and *a*_*ij*_ = 0 otherwise. For protein *i*, let *K*_*i*_ denote the connection degree of protein *i* (which is the sum of the *i*^*th*^ row or column in *G*). We can see the number of neighbors shared by protein *i* and *j* is

2.1 $$ {\sum}_{u\ne i,j}{a}_{iu}{a}_{uj} $$

So the connectivity between protein *i* and *j* through at most one inter media should be

2.2 $$ {\sum}_{u\ne i,j}{a}_{iu}{a}_{uj}+{a}_{ij} $$

To define a measure for the topological overlap, the above equation can be used as the numerator of the measure. For the denominator, it should satisfy two conditions: first, no less than the numerator, and second, greater than zero, so that the topological overlap should fall in [0,1].

In TOM, we define the denominator as *min*(*K*_*i*_, *K*_*j*_) + 1 − *a*_*ij*_. Since the elements in *G* is 0 or 1, so it’s easy to see that $\mathit{\min}\left({K}_i,{K}_j\right)+1-{a}_{ij}>\sum \limits_{u\ne i,j}{a}_{iu}{a}_{uj}+{a}_{ij}$, and *min*(*K*_*i*_, *K*_*j*_) + 1 − *a*_*ij*_ > 0. Finally, the TOM is defined as follows:

2.3 $$ TOM\left(i,j\right)=\frac{\sum_u{a}_{iu}{a}_{uj}+{a}_{ij}}{\mathit{\min}\left({k}_i,{k}_j\right)+1-{a}_{ij}} $$

The computation TOM based on *G* is straight forward. As we have defined the interaction between a protein to itself to be one, the numerator of TOM is *GG*^*T*^, the denominator is also a matrix, whose elements in position (*i*, *j*) should be $\sum \limits_u{a}_{iu}{a}_{uj}+1-{a}_{ij}$.

### The locational and topological overlap matrix model (LTOM)

**Fig. 2:**
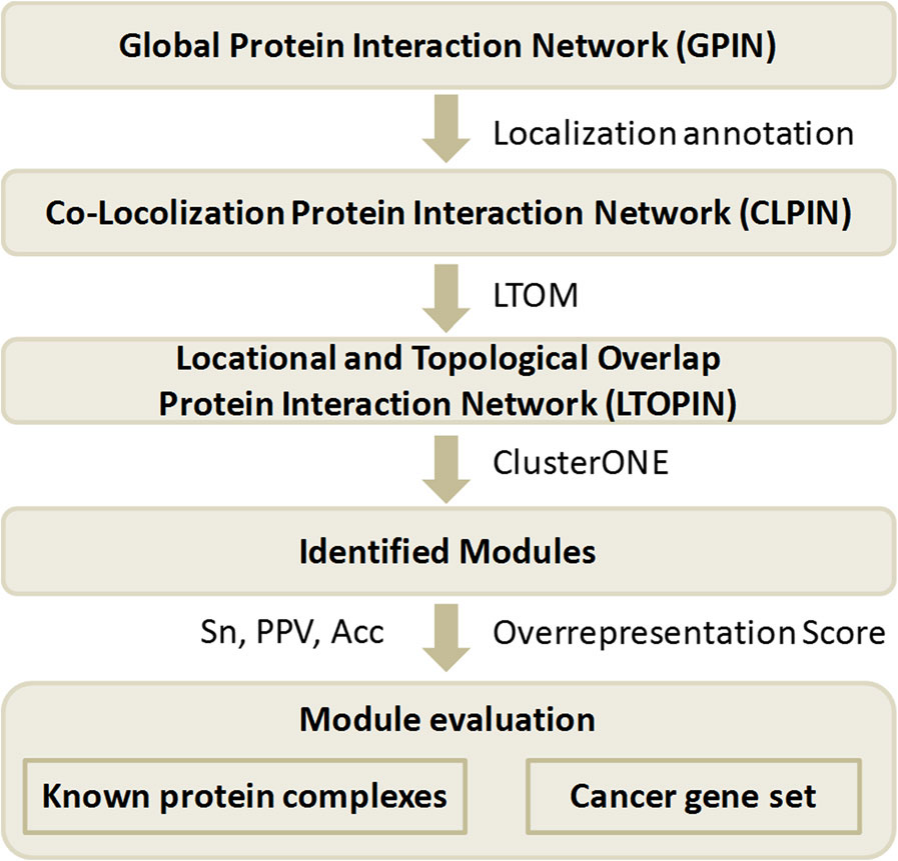
The locational and topological overlap model. A toy network is used to demonstrate the calculation steps. Nodes denote proteins while edges denote interactions. Different colors represent distinct subcellular localizations. Protein A and B share three overlapping partners. P1 and P3 have common localizations with both A and B whereas P2 does not. Specifically, the localizations of A-P2 and B-P2 are different (green edge vs brown edge)

The interaction information in TOM has been proved to be very useful and has been used in several published works [8, 27, 28]. In this paper, we aim at integrating the localization information into TOM to improve its power in detecting biological modules and named it as Locational and topological overlap matrix model (LTOM, see Fig. 2).

Suppose we have an additional localization matrix *L*, the rows of which represent proteins and columns represent locations. In *L*, its elements *b*_*ij*_ = 1 means the localization of protein *i* is *j*, and *b*_*ij*_ = 0 otherwise.

We maintain the denominator of LTOM the same as TOM, and multiply the numerator with a factor indicating the influence of the cell localization. Specifically, the LTOM is defined as:

2.4 $$ LTOM\left(i,j\right)=\frac{\sum_u{a}_{iu}{a}_{uj}\sigma \left(i,u,j\right)+{a}_{ij}{\sigma}^{\prime}\left(i,j\right)}{\mathit{\min}\left({k}_i,{k}_j\right)+1-{a}_{ij}} $$

where *σ* is a Boolean function indicting whether three proteins recorded in some same locations, since the connected two interactions of them are required to be in common locations in LTOM. In particular, function *σ*^′^ is defined on two proteins instead of three. The rest of the notations are defined the same as TOM.

Given the localization matrix *L*, *σ* and *σ*^′^ are easy to calculate. For *σ*^′^, it outputs 1 when 1 appears in the same column for two rows in matrix *L*, so ${\sigma}^{\prime}\left(i,j\right)=\mathit{\operatorname{sgn}}\left(\sum \limits_u{b}_{iu}{b}_{ju}\right)$. For *σ*, it outputs 1 when 1 appears in the same column for two rows in matrix *L*, so $\sigma \left(i,u,j\right)=\mathit{\operatorname{sgn}}\left(\sum \limits_k{b}_{ik}{b}_{uk}{b}_{jk}\right)$.

For the computation, please note that *σ*^′^ is a special case of *σ*, where two of the three indices are the same. So, the two parts in the numerator of LTOM can be calculated together and stored in a three-dimension matrix.

### Scale-free assessment

To assess whether a generated network has a scale-free topology, we used the power law distribution to fit the empirical data as follows,

2.5 $$ \mathrm{p}\left(\mathrm{k}\right)\sim {\mathrm{k}}^{-\upgamma} $$

where *k* is the degree of a given protein and *γ* is the degree exponent. Degree is defined as the number of partners that are connected to a given protein. *p*(*k*) refers to the probability that a protein has *k* interactions follows a power-law degree distribution. *γ* is larger than 0, where the smaller the value of *γ*, the larger fraction of the high-degree proteins [27, 28].

### Module identification and module benchmarks

Cluster with Overlapping Neighbourhood Expansion (ClusterONE) [7] was applied to identify protein modules for a series of protein interaction networks (PIN), including Global PIN (GPIN), Co-Localization PIN (CLPIN), Topological Overlap PIN (TOPIN) and Locational and Topological Overlap PIN (LTOPIN). ClusterONE is executed using a greedy growth algorithm to detect densely connected clusters from small seeds supervised by a fitness function. The quality of a cluster is evaluated by the number of internal interactions divided the number of theoretically possible interactions in the cluster. Given a module *V*, the number of interactions in the module is *w*^*in*^(*V*), and the number of interactions coming out of the module is *w*^*bound*^(*V*), then the score of cohesiveness is calculated as follows:

2.6 $$ f(V)=\frac{w^{in}(V)}{w^{in}(V)+{w}^{bound}(V)+p\mid V\mid } $$

where *p* ∣ *V* ∣ is a penalty term to model the network incompleteness considering the fact that a considerable number of interactions have not yet been detected. Modules are defined as the identified clusters with the size larger than 10 and network density larger than 0.25.

To evaluate the performance of module identification, we estimated the predicted modules to a reference set collected from Munich Information Center for Protein Sequences (MIPS) [29]. The latest version of the MIPS catalog of protein complexes was used as benchmarks in the study. The MIPS catalog was organized hierarchically and modules may involve submodules. We considered all MIPS categories with module size ranging from three to 100 as protein modules. MIPS category with 550 proteins and its descendants were excluded, since these are the predicted modules with low confidence.

Three measurements, clustering-wise sensitivity (*Sn*), positive predictive value (*PPV*), and accuracy (*Acc*) were used to evaluate the performance of different networks in identifying known protein complexes. The three measurements are defined as follow:

2.7 $$ \mathrm{Sn}=\frac{\sum_{\mathrm{j}=1}^{\mathrm{s}}{\max}_{\mathrm{i}=1,\dots \mathrm{r}}{\mathrm{t}}_{\mathrm{i}\mathrm{j}}}{\sum_{\mathrm{j}=1}^{\mathrm{s}}{\mathrm{w}}_{\mathrm{j}}} $$

2.8 $$ \mathrm{PPV}=\frac{\sum_{\mathrm{i}=1}^{\mathrm{r}}{\max}_{\mathrm{j}=1,\dots \mathrm{s}}{\mathrm{t}}_{\mathrm{i}\mathrm{j}}}{\sum_{\mathrm{i}=1}^{\mathrm{r}}{\sum}_{\mathrm{j}}^{\mathrm{s}}{\mathrm{t}}_{\mathrm{i}\mathrm{j}}} $$

2.9 $$ \mathrm{Acc}=\sqrt{\mathrm{Sn}\ast \mathrm{PPV}} $$

where *r* and *s* denote the number of predicted and known complexes, respectively, and *t*_*ij*_ denote the number of proteins in both of the predicted complex *i* and the reference complex *j*.

### Cancer proteins

We obtained the cancer genes from Cancer Gene Census (CGC, release v81) database [30]. It only collects cancer-associated genes with experimental evidence. This database contains 547 cancer-associated genes, among which 376 genes are annotated to the cell. The mapping from genes to proteins was curated between Ensemble gene ID and the UniProt Swiss-Prot accession number [21, 22]. Proteins encoded by cancer genes were simply referred to as cancer proteins.

### Enrichment analysis and the overrepresentation score

Hypergeometric test was used to test whether a module (a set of interesting proteins, say *M*) is overrepresented within a cancer gene set, say *X*.

2.10 $$ P={\sum}_{i=t}^n\frac{\left(\begin{array}{c}N-T\\ {}n-i\end{array}\right)\left(\begin{array}{c}T\\ {}i\end{array}\right)}{\left(\begin{array}{c}N\\ {}n\end{array}\right)} $$

where *N* is the number of proteins in PIN, *n* and *T* denote the module size of *M* and the size of *X*, respectively. *t* is the number of proteins in the module that is included in set *X*. *P* outputs the probability of observing *t* or more proteins of set *X* in module M of size n. It is then adjusted by the Benjamini & Hochberg False Discovery Rate (FDR) correction. We say cancer genes are enriched in module *M* if the adjusted *P* is less than a threshold of 0.01.

We use the Overrepresentation Score (ORS) [7] to measure the performance of our method to find *cancer modules* that are significantly enriched of cancer genes. ORS is the ratio of the number of cancer modules over the number of all the modules. Mathematically, it is defined as follows:

2.11 $$ ORS=\frac{\sum_i^U\mathit{\operatorname{sgn}}\left({P}_{cutoff}-{P}_{M_i}\right)}{U} $$

where *U* is the total number of predicted modules and ${P}_{M_i}$ represents the adjusted *P* value for module M and the cancer gene set. *P*_*cutoff*_ is the user-specified threshold of 0.01 by default.

## Results

### The co-localization protein interaction network has high confidence

Co-localization of the interacting proteins has provided an essential clue for their physical interaction. Here we conducted co-localization protein interaction networks (CLPIN) for HPRD [24] and BioGRID [25], respectively, in which all the interacting protein pairs are required to belong to at least one common location. Since all the information in the HPRD database has been manually extracted from the literature by expert biologists and it is frequently regarded as the reference of interactions, the proportion of co-localized protein pairs in HPRD is expected to be larger than the other PPI datasets. As shown in Table 1, the ratio of co-localized interaction in HPRD is as high as 62.46% (23,135/37,039) while the ratio of BioGRID is only 35.28% (94,780/268,684). The co-localized interactions are significantly overrepresented in the most reliable database (*p*-value < 2.2e-16, Hypergeometric Test), suggesting that the interacting protein pairs are prone to share the same subcellular localization. Not surprisingly, after co-localization filtering, interactions in the BioGRID database cover a significantly higher proportion of interactions in HPRD in comparison to the unfiltered dataset (data not shown).

**Table 1:** Network reliability comparison

Network	Protein	Interaction	Avg No. of literature	Verified in vivo	Precision	Recall	MCC
HPRD							
GPIN	8136	37,039	1.13	40.31%	0.8755	0.2058	0.2242
CLPIN	6882	23,135	1.23^a^	54.83% ^a^	0.9025	0.1900	0.2339
BioGRID							
GPIN	12,289	268,684	1.14	–	0.7842	0.2347	0.1741
CLPIN	9749	94,780	1.27^a^	–	0.8369	0.2211	0.2050

^a^Significant difference by RankSum test, *p* < 0.001. *PIN* Protein Interaction Network, *CLPIN* Co-Localization Protein Interaction Network. *Avg No. of literature* The average number of literature supported the interaction, *Verified in vivo* The percentage of protein interactions that have been verified in vivo in the HPRD database

Additionally, the interactions in CLPIN are well supported by literature (PubMed) and have significantly better experimental evidence. For HPRD, the average number of the supportting literature of the interactions in CLPIN is 1.23 whereas the number is 1.13 for GPIN (p-value < 2.2e-16, RankSum Test). Moreover, 54.83% (12,686/23,135) interactions out of the CLPIN have been verified in vivo, the ratio is also significantly higher than the counterpart (40.31%, 14,930/37,039) of the unfiltered interactions (p-value < 2.2e-16, Hypergeometric Test). For BioGRID, similarly, CLPIN has a significantly larger number of multiple publication-supported interactions with an average of 1.27, compared with the unfiltered GPIN of 1.14 (p-value < 2.2e-16, RankSum Test).

To assess the reliability of CLPIN, we also constructed a test set with positive and negative PPIs from another database Hippie (v2.1) [31]. Like Peng et al., we defined the interactions with top 5% highest confidence score as the positive set and randomly sampled the same number of interactions as the negative set [32]. As shown in Table 1, the scores of precision and MCC for CLPIN are higher than those of GPIN for both the HPRD and BioGRID datasets and the scores of recalls are comparable. These findings indicate that the protein pairs physically interacting with each other tend to localize in common cell compartments. In other words, the interacting partners annotated in the same location may have higher biological relevance.

### Construction of CLPIN, TOPIN, and LTOPIN

Since all the protein-protein interactions in HPRD are based on experimental evidence, we focus our analysis on HPRD. By combining the GPIN with localization annotation, we achieve a CLPIN covering 22,103 interactions that occur among 6794 human proteins. Topological Overlap PIN (TOPIN) and Locational and Topological Overlap PIN (LTOPIN) were constructed based on CLPIN, but only potential interactions were added in and no more external proteins are recruited, so they also contain 6794 proteins. After processed by the models of TOM and LTOM, a total of 26,473 and 25,007 interactions are included in TOPIN and LTOPIN, respectively.

Previous investigations have indicated that PIN possesses “scale-free” network features in different eukaryotic species, which also applied to other types of cellular networks, such as regulatory and metabolic networks [2–4]. Mathematically speaking, the “scale-free” property means the degrees of nodes in these networks follow a power law distribution. Biologically speaking, it suggests that there are a few highly connected nodes (also known as hubs) in the network, which are strongly associated with the biological function [27, 28]. To verify whether the four generated networks have a scale-free topology, we fit the power law distribution with empirical data for each of them. As shown in Table 2, we observed the frequency of the protein connectivity follows a power-law degree distribution, as the calculated degree exponents are all around 2.6. Although the number of interactions varies widely, these networks share similar degree distribution.

**Table 2:** Overview of the HPRD protein interaction networks

Network	Protein	Interaction	Average path length	Average clustering coefficient	Network density	Degree exponent
GPIN	7969	30,157	4.2425	0.1428	0.0009	2.6559
CLPIN	6794	22,103	4.4730	0.1539	0.0010	2.6118
TOPIN	6794	26,473	4.3919	0.2687	0.0012	2.6687
LTOPIN	6794	25,007	4.4165	0.2453	0.0011	2.6138

We have also systemically calculated other typical network parameters including network density, average shortest path length, and average clustering coefficient for the four networks. Since the TOPIN and LTOPIN are topologically extended on the CLPIN, the former two networks have relative larger networks with 26,473 and 25,007 interactions, respectively, as well as higher average clustering coefficients of 0.2687 and 0.2453 (Table 2). For the CLPIN, it is the smallest subnetwork with small average clustering coefficient (0.1539) and large average path length (4.4730). All of these results indicate that compared with the CLPIN, the TOPIN and LTOPIN networks contain more proteins with high connectivity. On the contrary, the network parameters such as density and clustering coefficient for GPIN are consistently low, suggesting that the overall connection in the network is relatively sparse and proteins in this network are less prone to form modules.

### Performance comparison on protein complexes

Protein module prediction is one of the most typical applications for protein interaction network. As illustrated in Fig. 1, we used ClusterONE to identify functional modules from GPIN, CLPIN, TOPIN, and LTOPIN, respectively. 10, 8, 51, and 34 modules are separately identified with module size greater than ten using these four networks. To assess the performance of module identification, we considered the complexes included in MIPS as the benchmark and a total of 932 complexes of sizes no less than three were defined as the reference. Our finding shows that LTOPIN outperforms the other networks in identifying modules regarding the three evaluation measurements, the clustering-wise sensitivity (*Sn*), positive predictive value (*PPV*), and their geometric accuracy (*Acc*) (see methods). Specifically, in Table 3, the LTOPIN inferred module set has the maximum *Sn* of 0.1354 and *Acc* of 0.1424, although the *PPV* (0.1497) is the second highest. Similar results can be obtained when identifying modules with other thresholds of module size ranging from 5 to 9 (Table 3 and Additional file 1: Table S1). These findings demonstrate that the clustering-wise sensitivity can be significantly improved by applying the LTOM model with the trade-off of *PPV*.

**Table 3:** Performance comparison on known protein complexes

PIN	Module Number	module size > = 5			Module Number	module size > = 10		
		Sn	PPV	ACC		Sn	PPV	ACC
HPRD								
GPIN	336	0.277	0.2143	0.2436	10	0.081	0.1259	0.101
CLPIN	252	0.2488	0.2095	0.2283	8	0.0691	0.1207	0.0913
TOPIN	376	0.2981	**0.2144**	0.2528	51	0.1109	**0.1754**	0.1395
LTOPIN	355	**0.3292**	0.1966	**0.2544**	34	**0.1354**	0.1497	**0.1424**
Yeast								
GPIN	614	0.6148	**0.5984**	0.6066	112	0.5495	**0.5504**	0.55
CLPIN	344	0.717	0.5592	0.6332	106	0.5939	0.5046	0.5474
TOPIN	492	0.7151	0.5678	0.6372	143	0.5977	0.5124	0.5534
LTOPIN	490	**0.7456**	0.5663	**0.6498**	141	**0.6256**	0.5104	**0.5651**

Modules were identified using ClusterONE with module size no less than five and ten, respectively. Bold values denote the best scores corresponding to specific criteria. *Sn* Sensitivity, *PPV* The positive predictive value, *ACC* The geometric accuracy

In addition, the same conclusion can be drawn using a yeast PPI dataset with good completeness. The yeast dataset has broader coverage of interactions than HPRD and the inferred modules score higher across all network models, i.e., GPIN, CLPIN, TOPIN, and LTOPIN. As shown in Table 3, the LTOPIN inferred module set achieves the highest sensitivity and accuracy, 0.6256 for *Sn* and 0.5651 for *Acc,* respectively. Loosening the module size threshold to five, more modules are produced and all the evaluation scores are improved substantially in each PIN, while LTOPIN still outperforms the others. In particular, the *Acc* is as high as around 0.65 for the LTOPIN-modules, whose *Sn* (around 0.75) is the highest across all the produced networks. Overall, in comparison with the GPIN, CLPIN, and TOPIN, the LTOPIN consistently has a superior performance in identifying known protein complexes, indicating that the LTOM model helps reveal the biological relation over interacted proteins.

### The LTOPIN inferred modules overrepresent cancer genes

To further detect the biological functions of the predicted modules, we associated these modules with human cancer genes, since accumulated evidences have shown that proteins encoded by cancer genes tend to work together as modules to execute their functions [3, 33, 34]. As expected, we found that cancer proteins are more likely to be involved in the extended-network inferred modules, TOPIN and LTOPIN, when compared with the modules generated from other networks, GPIN and CLPIN. Table 4 shows that cancer genes are strikingly enriched within the modules of TOPIN and LTOPIN, 49.02 and 44.10% among them involve cancer genes, whereas the ratios are 25% for CLPIN and 20% for GPIN, respectively. When using a stricter measurement, overrepresentation score (ORS, defined in Eq. 2.11), LTOPIN-modules achieves the highest ORS of 0.1765, suggesting the LTOM model is able to produce more cancer modules that are overrepresented cancer genes.

**Table 4:** Overrepresentation scores of cancer genes in HPRD

PIN	Module number	Cancer module ratio	ORS	Cancer gene ratio (number)
module size > = 10				
GPIN	10	0.2	0	0 (0)
CLPIN	8	0.25	0.125	0.0133 (6)
TOPIN	51	0.4902	0.098	0.0399 (18)
LTOPIN	34	0.441	0.1765	0.0421 (19)
module size > = 5				
GPIN	336	0.273	0.0504	0.0887 (40)
CLPIN	252	0.3004	0.0593	0.0931 (42)
TOPIN	376	0.3528	0.0504	0.1242 (56)
LTOPIN	355	0.3539	0.0702	0.1441 (65)

*ORS* Overrepresentation Score, *Cancer module ratio* The ratio of modules containing cancer genes, *Cancer gene ratio* The ratio of cancer genes over genes in cancer modules

Please note that ClusterONE may produce overlapping modules and some cancer genes may appear in more than one module. To avoid double counting, we also calculated the ratio (number) of cancer genes involved in the cancer modules. As shown in Table 4, the LTOPIN produced cancer modules contain a total of 19 cancer genes (4.21%), which is slightly higher than the cancer modules of TOPIN (18) and much higher than the other two (0 and 6). The advantages of LTOM are even more apparent when focusing on modules larger than five (Table 4). The LTOPIN inferred cancer modules involve 65 unique cancer genes, accounting for 14.41% of the entire cancer gene list, which is much higher than the others (12.42, 9.31, and 8.87%, respectively). These findings suggest that the LTOM imputed networks are able to identify modules that are more relevant to cancer genes when compared with the traditional model of TOM and other non-modeled networks.

## Conclusion

In this paper, we firstly demonstrated that the co-localized interacting proteins have higher experimental confidence in their interactions. Then, we proposed a Locational and Topological Overlap Model (LTOM) to impute the potential interactions taking both the locational and topological information of proteins into account. The model was demonstrated to improve the performance of module identification for the raw protein interaction networks of human and yeast.

In general, a protein module in a network should be the one that has dense interactions between the inside proteins and well-separated from the outside proteins. ClusterONE models the properties using the cohesiveness score and can identify overlapping protein modules from the PPI networks by the guidance of cohesiveness. That is why we use ClusterONE to identify modules. However, LTOM is a general imputation step of protein interaction networks that can be embedded in any appropriate module identification approach depending on the user preferences. So, another commonly used clustering method, Markov Clustering Algorithm (MCL), was also applied to the processed networks and its performance was improved considerably when using LTOM (Additional file 1: Table S2). Overall, our results show that LTOM constantly improves the performance of existing clustering methods for protein module prediction. In addition to the protein interaction networks, the model can readily be used for the other types of cellular networks, such as gene coding-non-coding co-expression networks, RNA-protein regulatory networks, or RNA-RNA interaction networks [35–41].

## Additional file

Additional file 1: **Table S1.** Performance comparison on known protein complexes using ClusterONE. **Table S2.** Performance comparison on known protein complexes using MCL. (DOCX 22 kb)

## Abbreviations

Acc: Accuracy
CLPIN: Co-localization PIN
FDR: False Discovery Rate
GPIN: Global PIN
LTOM: Locational and Topological Overlap Model
LTOPIN: Locational and Topological Overlap PIN
MCL: Markov Clustering Algorithm
ORS: Overrepresentation Score
PIN: Protein Interaction Network
PPV: Positive predictive value
Sn: Clustering-wise sensitivity
TOM: Topological Overlap Matrix
TOPIN: Topological Overlap PIN
