# Supplementary material for: Exploiting locational and topological overlap model to identify modules in protein interaction networks
Source: BMC Bioinformatics. 2019 Jan 14;20:23. doi: 10.1186/s12859-019-2598-7 (PMC6332531; doi:10.1186/s12859-019-2598-7)
Supplement: Supplementary file 1 — Table S1. Performance comparison on known protein complexes using ClusterONE. Table S2. Performance comparison on known protein complexes using MCL. (DOCX 22 kb) [file 12859_2019_2598_MOESM1_ESM.docx]

**Additional file 1:**

**Table S1** Performance comparison on known protein complexes using ClusterONE.

|  | **HPRD** | | | | **Yeast** | | | |
| --- | --- | --- | --- | --- | --- | --- | --- | --- |
|  | Module Number | Sn | PPV | Acc | Module Number | Sn | PPV | Acc |
| Module Size >=6 | | | | | | | | |
| GPIN | 130 | 0.2079 | 0.1866 | 0.1970 | 316 | 0.5984 | **0.5754** | 0.5868 |
| CLPIN | 114 | 0.1897 | 0.1860 | 0.1879 | 228 | 0.6872 | 0.5541 | 0.6170 |
| TOPIN | 237 | 0.2574 | **0.1950** | 0.2240 | 363 | 0.6948 | 0.5574 | 0.6223 |
| LTOPIN | 210 | **0.2870** | 0.1758 | **0.2246** | 363 | **0.7297** | 0.5544 | **0.6360** |
| Module Size >=7 | | | | | | | | |
| GPIN | 44 | 0.1495 | 0.164 | 0.1565 | 171 | 0.5755 | **0.5627** | 0.5691 |
| CLPIN | 41 | 0.1451 | 0.164 | 0.1543 | 158 | 0.6618 | 0.5389 | 0.5972 |
| TOPIN | 126 | 0.1897 | **0.1785** | 0.1840 | 238 | 0.6624 | 0.5407 | 0.5985 |
| LTOPIN | 103 | **0.2257** | 0.166 | **0.1936** | 239 | **0.6999** | 0.5383 | **0.6138** |
| Module Size >=8 | | | | | | | | |
| GPIN | 27 | 0.1328 | 0.1455 | 0.1390 | 146 | 0.5660 | **0.556** | 0.5610 |
| CLPIN | 25 | 0.1178 | 0.1481 | 0.1320 | 141 | 0.6396 | 0.5303 | 0.5824 |
| TOPIN | 89 | 0.1620 | **0.1755** | 0.1686 | 198 | 0.6352 | 0.5283 | 0.5793 |
| LTOPIN | 75 | **0.2091** | 0.1538 | **0.1793** | 195 | **0.6694** | 0.5232 | **0.5918** |
| Module Size >=9 | | | | | | | | |
| GPIN | 14 | 0.1058 | 0.1409 | 0.1221 | 125 | 0.5615 | **0.5541** | 0.5578 |
| CLPIN | 12 | 0.0970 | 0.1381 | 0.1158 | 117 | 0.6136 | 0.5189 | 0.5643 |
| TOPIN | 61 | 0.1401 | **0.1725** | 0.1554 | 159 | 0.6129 | 0.5186 | 0.5638 |
| LTOPIN | 48 | **0.1808** | 0.1543 | **0.1670** | 157 | **0.6402** | 0.5189 | **0.5764** |

Modules were identified using ClusterONE with module size threshold ranging from 6 to 9. Bold values denote the best scores corresponding to specific criteria. Sn, sensitivity; PPV, the positive predictive value; ACC: the geometric accuracy.

**Table S2** Performance comparison on known protein complexes using MCL.

|  | | Module Number | Sn | PPV | Acc |
| --- | --- | --- | --- | --- | --- |
| Module Size >=5 |  | |  |  |  |
| GPIN | | 412 | 0.3518 | 0.1421 | 0.2236 |
| CLPIN | | 323 | 0.3348 | **0.1437** | 0.2193 |
| TOPIN | | 312 | 0.3615 | 0.1429 | 0.2272 |
| LTOPIN | | 308 | **0.3914** | 0.143 | **0.2366** |
| Module Size >=10 | |  |  |  |  |
| GPIN | | 151 | 0.291 | 0.1124 | 0.1808 |
| CLPIN | | 125 | 0.2846 | 0.1078 | 0.1752 |
| TOPIN | | 125 | 0.2964 | **0.1146** | 0.1843 |
| LTOPIN | | 131 | **0.3356** | 0.1114 | **0.1933** |

Modules were identified using MCL with module size no less than five and ten, respectively, from the HPRD dataset. Jaccard coefficient thresholds were set as 0.5. Bold values denote the best scores corresponding to specific criteria.
